# Host–microbial interactions differ with age of asthma onset

**DOI:** 10.1183/13993003.00428-2024

**Published:** 2024-09-05

**Authors:** Ali Versi, Adnan Azim, Fransiskus Xaverius Ivan, Mahmoud I. Abdel-Aziz, Stewart Bates, John Riley, Anke H. Maitland-Van Der Zee, Sven-Erik Dahlen, Ratko Djukanovic, Sanjay H. Chotirmall, Peter Howarth, Nazanin Zounemat Kermani, Kian Fan Chung, Ian M. Adcock

**Affiliations:** 1 National Heart and Lung Institute and Data Science Institute, Imperial College London, London, UK; 2 Faculty of Medicine, Southampton University, Southampton, UK; 3 Lee Kong Chian School of Medicine, Nanyang Technological University, Singapore, Singapore; 4 Amsterdam University Medical Centers, Department of Pulmonary Medicine, University of Amsterdam, Amsterdam, The Netherlands; 5 Respiratory Therapeutic Unit, GSK, Stockley Park, UK; 6 Department of Medicine Huddinge, Karolinska Institutet, Stockholm, Sweden; 7 Department of Respiratory and Critical Care Medicine, Tan Tock Seng Hospital, Singapore, Singapore

## Abstract

Asthma is a heterogenous disease [1] and dichotomisation between childhood/early-onset (EO) and adult/late-onset (LO) disease [2] identified differences in lung function decline and response to anti-inflammatory therapies, including biologics [3]. This suggests distinct inflammatory mechanisms underpin EO and LO asthma. In parallel, a relationship exists between airway neutrophilia and the airway microbiome [4, 5]. We postulate that differences in host–microbial interactions are associated with the age of asthma onset and would be maintained over time. Here, we applied a recently described machine learning framework, sparse canonical correlation analysis (Sparse-CCA) [6], to identify differences in host–microbial interactions in the airways of EO and LO asthma.

## Shareable abstract

**The host immune response to pathogens differs between early- and late-onset asthma** https://bit.ly/3xSa4Fe

*To the Editor:*

Asthma is a heterogenous disease [1] and dichotomisation between childhood/early-onset (EO) and adult/late-onset (LO) disease [2] identified differences in lung function decline and response to anti-inflammatory therapies, including biologics [3]. This suggests distinct inflammatory mechanisms underpin EO and LO asthma. In parallel, a relationship exists between airway neutrophilia and the airway microbiome [4, 5]. We postulate that differences in host–microbial interactions are associated with the age of asthma onset and would be maintained over time. Here, we applied a recently described machine learning framework, sparse canonical correlation analysis (Sparse-CCA) [6], to identify differences in host–microbial interactions in the airways of EO and LO asthma.

The U-BIOPRED cohort is a severity-based cross-sectional study of asthma in Europe whose inclusion and exclusion criteria have been previously described [7]. We undertook a combined analysis of sputum transcriptomics (Affymetrix U133 Plus microarrays using RNA from sputum cells) [8] and sputum metagenomics (Illumina HiSeq® 2500 platform) [9]. The number of paired samples consisted of 79 subjects with severe asthma that included non-smokers and current and/or ex-smokers, and 20 with mild–moderate asthma. These paired samples were divided into EO (n=43) and LO (n=56) asthma using 18 years of age as the threshold (figure 1a). There was no significant difference between the clinical characteristics of the subjects included here and of the whole U-BIOPRED cohort.

**FIGURE 1:**
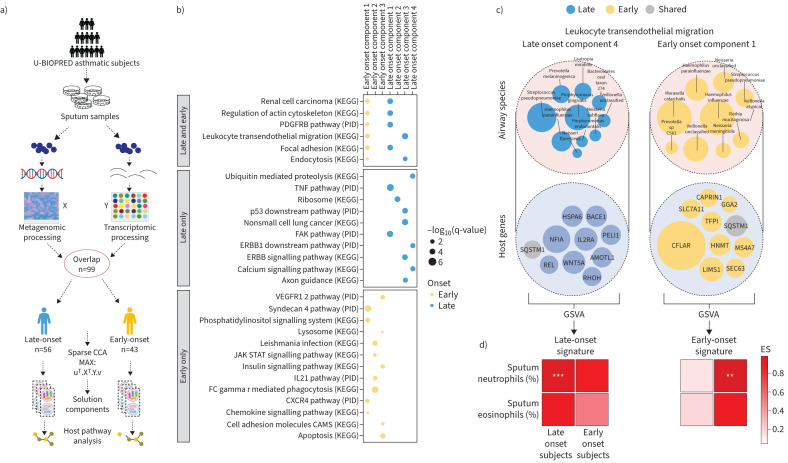
a) Flowchart showing the overall method of sample collection, cohort grouping and the application of sparse canonical correlation analysis (Sparse-CCA). b) Component genes pathway analysis showing the enrichment of pathways for each component. c) Visualisation of leukocyte transendothelial migration for both adult and child onset showing the top 10 genes and species by absolute weights. d) Gene set variation analysis (GSVA) sample-wise enrichment score (ES) correlation with clinical characteristics showing the correlation of the ES of each gene set from the components in the leukocyte transendothelial migration pathway with sputum neutrophils and eosinophils. KEGG: Kyoto Encyclopaedia of Genes and Genomes; PID: pathway interaction database; TNF: tumour necrosis factor. Significance of Spearman coefficient correlation: not significant (p>0.05) indicated by clear box; **: p≤0.01; ***: p≤0.001.

The median (interquartile range) age of onset of LO patients (39.0 (27.0–50.5) years) was significantly different from that of EO patients (5.0 (3.0–10.0) years; p<0.001). There was a significant difference (p<0.001) in the mean±sd duration of asthma (age at recruitment minus age of asthma onset) between the LO (16.7±12.2 years) and EO (36.3±14.0 years) groups. The age at which samples from the patients in the LO group (57.5 (51.0–64.2) years) samples were collected was significantly different from the age at which EO patient samples were collected (46.0 (32.5–52.0) years; p<0.001) suggesting that age may be an important factor when considering host–microbiome interactions in asthma.

LO patients had significantly higher blood (0.4±0.3 *versus* 0.3±0.2×10^9^ L^−1^; p<0.05) and sputum eosinophils (15.8±21.1 *versus* 6.6±13.5%; p<0.05) than EO patients, suggesting a greater type 2 inflammatory drive. In addition, LO subjects had a significantly greater prevalence of subjects with nasal polyposis compared to EO patients (44.6 *versus* 20.9%; p<0.05). In contrast, EO subjects were more atopic (93.0 *versus* 58.9%; p=0.001) as defined by either skin prick testing or measurement of specific IgE to six common aeroallergens [7], with a greater number of subjects with allergic rhinitis (55.8 *versus* 30.4%; p<0.05) and hay fever (65.1 *versus* 41.1%; p<0.05) compared to LO subjects. There were no significant differences in sputum neutrophils, sputum macrophages age, lung function (forced expiratory volume in 1 s), exhaled nitric oxide fraction, body mass index, oral corticosteroid use, Asthma Control Questionnaire 5 scores, allergic comorbidities, sex, exacerbations, antibiotic use, blood granulocyte levels and serum biomarkers between groups. 48.2% LO subjects were non-smokers, compared to 79.1% in the EO group (p<0.005). There was no significant difference in smoking history (13.5±16.8 *versus* 12.3±9.6 pack years) or current smoking status between groups.

4405 differentially expressed genes (DEGs, adjusted p-value <0.05 and a log_2_ fold change (FC) >0.5) were identified between severe asthma and healthy control subjects (n=79 and 23 respectively). Genes with low variance were filtered using a 25% quantile of variance across each group, leaving 3304 DEGs. After this preprocessing, the severe asthma (n=79) and mild–moderate asthma cohorts (n=20) were used in the Sparse-CCA to study EO *versus* LO disease. For the metagenomics dataset, abundance-based filtering was performed to retain species found at 0.01 relative abundance in at least 10% of samples across each group.

Sparse-CCA incorporates a lasso (Least Absolute Shrinkage and Selection Operator) penalty for feature selection and a linear projection of two sets of observations into a shared latent space [6] which identifies a smaller subset of paired host genes and bacterial species, known as components that are most highly correlated for each age-of-onset-group. The analysis was conducted using R-4.1.3 with the PMA package (version 1.2.1). Hyperparameter tuning was performed using a grid search approach to identify parameters for sparsity penalties, as previously described [6]. Sparse CCA components were computed for each group with no components being correlated with each other.

Sparse-CCA generated five components in LO asthma (LOC1–5) and three in EO asthma (EOC1–3) (figure 1b). Three out of five components in LO asthma (LOC1, 2, 4) and two of three components in EO asthma (EOC1, 3) correlated (Spearman's) with sputum neutrophilia (LOC1 r=0.36, p<0.01; LOC2 r=0.70, p<0.001; LOC4 r=0.35, p<0.01; EOC1 r=0.36, p<0.05 and EOC3 r=0.63, p<0.001), demonstrating the salience of these host–microbial interactions on clinical phenotype.

Pathway enrichment analysis of the Kyoto Encyclopaedia of Genes and Genomes (KEGG) and pathway interaction database (PID) gene sets from the MsigDB canonical pathways collection (https://www.gsea-msigdb.org/gsea/msigdb/human/collections.jsp) across all components identified six pathways that were shared between phenotypes. In addition, 10 pathways were only enriched in LO asthma and 13 pathways only enriched in EO asthma according to absolute weighting using a Fisher's exact test (p<0.05).

Compared to LO asthma or pathways shared between LO and EO asthma, there was greater enrichment of pathways associated with adhesion molecules in the components of EO asthma. This is consistent with genetic studies that identify barrier function as a contributor to EO disease [10] as well as its greater association with atopic conditions [11] (figure 1b). In addition, PID pathways specific for EOC1 were associated with cell adhesion, migration and proliferation, whilst KEGG pathways in EOC2 are linked to pathways involved in immune signalling in response to microbial infection. Finally, EOC3 is associated with cell proliferation/death pathways and insulin resistance (figure 1b).

Sparse-CCA identified tumour necrosis factor (TNF) signalling to be more prominent in LOC1 (figure 1b). TNF is a pro-inflammatory cytokine associated with neutrophilic asthma [12]. While direct therapeutic targeting of TNF in asthma has not been successful, azithromycin therapy modulates the TNF axis [13]. Integrin- and other cell surface receptor-mediated intracellular signalling (FAK pathway), ribosome and gene expression, proliferative pathways and calcium signalling pathways were associated with LOC2–5, respectively (figure 1b).

Several pathways were shared between LO and EO phenotypes, particularly EOC1 and LOC2 and 4 (figure 1b). However, the genes and species constituting those components were not identical; for example, LOC4 and EOC1 were both enriched for leukocyte transendothelial migration (figure 1b and c), but LOC4 was characterised by *Streptococcus species* and Wnt5a while EOC1 was characterised by *Moraxella catarrhalis, Haemophilus influenzae* and CFLAR (figure 1c). SQSTM1, present in both components, modulates microbe-induced inflammatory pathways in an autophagy-dependent and -independent manner [14]. The combination of Wnt5a and CFLAR (figure 1c) with SQSTM1, for example, on microbial growth and on host–microbe immune interactions should be the target of further studies.

Using gene set variation analysis (GSVA) to calculate a sample-wise enrichment score (ES) from the top 10 genes in a component by absolute weights from the Sparse-CCA, genes in LOC4 correlated with sputum neutrophilia in LO patients but not in EO asthma. Conversely, genes in EOC1 correlated with sputum neutrophilia in EO but not LO asthma (figure 1d). These findings indicate that these host-microbial interactions are unique to each neutrophilic asthma phenotype.

*Haemophilus influenzae* was associated with pathways enriched in both EO and LO components (15 in EO and 10 in LO), *Moraxella catarrhalis* was only associated with pathways enriched in EO (19 pathways) and *Tropheryma whipplei* with pathways enriched in LO (6 pathways). LOC3 had a geneset, whose ES was correlated with sputum eosinophils and was dominated by *Neisseria* and *Haemophilus influenza*.

In summary, sparse-CCA identified several host gene–microbiome associations; however, longitudinal/dynamic conclusions regarding causality cannot be inferred in this cross-sectional/static analysis. Furthermore, exposures cannot be accounted for and will clearly influence disease evolution, and a temporal microbiomics approach may be required to identify underlying endotypes [15]. Moreover, U-BIOPRED is an adult cohort and so age of disease onset is confounded by disease duration, number of exacerbations and treatment including corticosteroids and macrolides. Nevertheless, microbial analysis of the U-BIOPRED data has previously identified differences in microbial profiles and age of onset [4]. This analysis extends those findings by identifying shared and unique host-microbial interactions between EO and LO phenotypes.

This study demonstrates the utility of integrating the sputum microbiome and host gene expression together to obtain insight into their contribution to the disease process, which is superior to single-dataset omics alone. While the composition of the airway microbiome changes throughout life, it is particularly dynamic in the early years of life, when perturbations are thought to be critical to lower airway immune maturation [16]. Our findings demonstrate that the heterogeneity of asthma immunopathophysiology may be better understood though host–microbial interactions.

## Shareable PDF

10.1183/13993003.00428-2024.Shareable1 This one-page PDF can be shared freely online.
Shareable PDF ERJ-00428-2024.Shareable

## Data Availability

Individual and group data will be made available immediately after publication and after ensuring de-identification. The study protocol (NCT01976767), informed consent and cohort clinical data have been published previously [7]. The sputum transcriptomics data is available at GSE76262. Metagenomics data will be made available after reasonable written request to the U-BIOPRED consortia management.
