# Supplementary material for: Host–microbial interactions differ with age of asthma onset
Source: Eur Respir J. 2024 Sep 5;64(3):2400428. doi: 10.1183/13993003.00428-2024 (PMC11375513; doi:10.1183/13993003.00428-2024)
Supplement: Supplementary file 1 [file ERJ-00428-2024.Shareable.pdf]

EUROPEAN RESPIRATORY JOURNAL
RESEARCH LETTER
A. VERSI ET AL.

SHAREABLE PDF

Host–microbial interactions differ with age of asthma onset

Ali Versi[1], Adnan Azim [2], Fransiskus Xaverius Ivan[3], Mahmoud I. Abdel-Aziz [4], Stewart Bates[5], John Riley[5], Anke H. Maitland-Van Der Zee[4], Sven-Erik Dahlen[6], Ratko Djukanovic[2], Sanjay H. Chotirmall [3,7], Peter Howarth [2], Nazanin Zounemat Kermani[1], Kian Fan Chung[1] and Ian M. Adcock [1], on behalf of the U-BIOPRED study group

[1]National Heart and Lung Institute and Data Science Institute, Imperial College London, London, UK. [2]Faculty of Medicine, Southampton University, Southampton, UK. [3]Lee Kong Chian School of Medicine, Nanyang Technological University, Singapore, Singapore. [4]Amsterdam University Medical Centers, Department of Pulmonary Medicine, University of Amsterdam, Amsterdam, The Netherlands. [5]Respiratory Therapeutic Unit, GSK, Stockley Park, UK. [6]Department of Medicine Huddinge, Karolinska Institutet, Stockholm, Sweden. [7]Department of Respiratory and Critical Care Medicine, Tan Tock Seng Hospital, Singapore, Singapore.

Corresponding author: Ian M. Adcock (ian.adcock@imperial.ac.uk)

Shareable abstract (@ERSpublications)
The host immune response to pathogens differs between early- and late-onset asthma https://bit.ly/3xSa4Fe

Cite this article as: Versi A, Azim A, Ivan FX, *et al.* Host–microbial interactions differ with age of asthma onset. *Eur Respir J* 2024; 64: 2400428 [DOI: 10.1183/13993003.00428-2024].

This extracted version can be shared freely online.

Copyright ©The authors 2024.

This version is distributed under the terms of the Creative Commons Attribution Licence 4.0. For reproduction rights and permissions contact permissions@ersnet.org

Received: 29 Feb 2024
Accepted: 2 July 2024

To the Editor:

Asthma is a heterogenous disease [1] and dichotomisation between childhood/early-onset (EO) and adult/late-onset (LO) disease [2] identified differences in lung function decline and response to anti-inflammatory therapies, including biologics [3]. This suggests distinct inflammatory mechanisms underpin EO and LO asthma. In parallel, a relationship exists between airway neutrophilia and the airway microbiome [4, 5]. We postulate that differences in host–microbial interactions are associated with the age of asthma onset and would be maintained over time. Here, we applied a recently described machine learning framework, sparse canonical correlation analysis (Sparse-CCA) [6], to identify differences in host–microbial interactions in the airways of EO and LO asthma.
